# Can digital stories go where palliative care research has never gone before? A descriptive qualitative study exploring the application of an emerging public health research method in an indigenous palliative care context

**DOI:** 10.1186/s12904-017-0216-x

**Published:** 2017-09-04

**Authors:** Lisa Williams, Merryn Gott, Tess Moeke-Maxwell, Stella Black, Shuchi Kothari, Sarina Pearson, Tessa Morgan, Matua Rawiri Wharemate, Whaea Whio Hansen

**Affiliations:** 1 School of Nursing, Faculty of Medical and Health Sciences, University of Auckland, Auckland, New Zealand; 2 Media and Communication, School of Social Sciences, Faculty of the Arts, University of Auckland, Auckland, New Zealand; 3 Kaumātua, Te Ārai: Palliative Care and End of Life Research Group, School of Nursing, Faculty of Medical and Health Sciences, University of Auckland, Auckland, New Zealand

**Keywords:** Palliative care, End of life care, Māori, Indigenous palliative care, Research methods, Digital storytelling, Kaupapa Māori

## Abstract

**Background:**

The World Health Organization (WHO) has called for global approaches to palliative care development. Yet it is questionable whether one-size-fits-all solutions can accommodate international disparities in palliative care need. More flexible research methods are called for in order to understand diverse priorities at local levels. This is especially imperative for Indigenous populations and other groups underrepresented in the palliative care evidence-base.

Digital storytelling (DST) offers the potential to be one such method. Digital stories are short first-person videos that tell a story of great significance to the creator. The method has already found a place within public health research and has been described as a useful, emergent method for community-based participatory research.

**Methods:**

The aim of this study was to explore Māori participants’ views on DST’s usefulness, from an Indigenous perspective, as a research method within the discipline of palliative care. The digital storytelling method was adapted to include Māori cultural protocols. Data capturing participant experience of the study were collected using participant observation and anonymous questionnaires. Eight participants, seven women and one man, took part. Field notes and questionnaire data were analysed using critical thematic analysis.

**Results:**

Two main themes were identified during analyses: 1) issues that facilitated digital storytelling’s usefulness as a research method for Māori reporting on end of life caregiving; and 2) issues that hindered this process. All subthemes identified: recruitment, the pōwhiri process, (Māori formal welcome of visitors) and technology, related to both main themes and are presented in this way.

**Conclusion:**

Digital storytelling is an emerging method useful for exploring Indigenous palliative care issues. In line with a Health Promoting Palliative Care approach that centres research in communities, it helps meet the need for diverse approaches to involve underrepresented groups.

## Background

It is well established that populations are ageing [1, 2]. In tandem, there is and will continue to be a growing need for palliative care due to the concurrent rise in non-communicable diseases [2, 3]. In response to the rapidly growing need for palliative care, the World Health Organization (WHO) has called for global approaches to palliative care development [3]. However, this one-size-fits-all outlook has recently been challenged [4]. Citing cultural, logistical, infrastructural and economic disparities amongst the countries of the world, Zaman et al. question whether localised alternatives might be more effective [4]. They offer the contested notion of the ‘good death’ as one example. Dissimilarities in religious and spiritual expectations around death, appropriate place of death and views on euthanasia or assisted dying problematise a simple, unified definition of what a ‘good death’ could possibly mean [4].

Among Zaman et al.’s [4] suggestions for countering a homogenised perspective on palliative care is that research draws upon ‘culturally and historically informed methods.’ ^p.77^ This fits with a recent move towards adopting a public health approach to palliative care which prioritises community perspectives and cautions against relying too heavily on professionally-led solutions [5]. Indeed, approaches such as Health Promoting Palliative Care [6], acknowledge the centrality of community engagement initiatives, especially for marginalised groups [5]. Speaking specifically about issues of continuity of care and equity, the authors assert that developing sustainable community support structures via community engagement may be a way to juxtapose ‘discrete episodes of professional intervention’ with continuous support centred in the community [5]. ^(p. 233)^

Community-centred palliative care approaches call for community-centred research methods, especially ones that may be adapted for the particularities of locality. Digital storytelling (DST) offers the potential to be one such method. Originally developed by the Story Center in Berkeley, California [7], digital stories are short, first-person videos comprised of images, video clips, voiceovers, text and music that form a narrative about a story of great significance to the teller. They are developed over the course of a few days in a workshop setting led by leaders who offer instruction and support on how to craft participant’ stories into scripts and then develop them into finished videos [8]. A significant component of the workshop is the story circle in which participants share their story ideas and receive feedback from the group before moving on to writing scripts.

DST has already found a place within public health. Gubrium describes its usefulness for community based participatory research (CBPR) regarding health promotion and practice [9]. Gubrium and DiFulvio use digital stories to explore Latina girls’ lived experiences around issues of health and wellbeing [10]. Cueva et al.’s research looks at how digital stories about cancer influenced community health aides knowledge, attitudes and health behaviours and how they used their stories as health communication tools [11].

Given the method’s promise, we believed it imperative to explore how DST might be applied within the discipline of palliative care. The reason for our specific locus of inquiry, Māori experiences of caring for their older relatives at the end of life, arose because Māori, like Indigenous groups internationally, are underrepresented in the palliative care literature. Most Indigenous-specific research has only appeared within the last 15 years; [12, 13] it was not until 2006 that a specific Māori perspective on palliative care emerged [14].

However, it cannot be assumed that simply applying a method developed for western audiences will be suitable for Indigenous groups [15]. Researchers need to adopt or adapt methods that do not repeat historical cultural domination. Referring to western researchers’ engagement with Māori, Smith [15] states that due to past deleterious encounters, many Māori had come to view western researchers as ‘simply intent on taking or ‘stealing’ knowledge in a non-reciprocal and often underhanded way’.^(p. 289)^ Pacheco [16] asserts that American Indians’ mistrust ‘is grounded in repeated, well-documented examples of unethical medical research and clinical misconduct in the name of research.’^(p.2153)^ Australian Aborigines share a similar viewpoint; Guillemin documents how they started from ‘a position of caution and distrust’ when engaging with researchers [17].^(p.7)^

Such mistrust can be ameliorated by research methods that foster partnerships with Indigenous communities. For example, in Canada, the persistence of health inequality amongst Indigenous and non-Indigenous people led to a call for new approaches to research that established communities as co-creators of research so that their concerns could take center stage [18]. In the United States, the Center for American Indian Community Health (CAICH) used Community Based Participatory Research (CBPR) to promote health equity among American Indians. Pacheco cites the organization’s 8-year track record as proof of their success; its participatory research and education programme led to partnerships between academics, community organisations and tribes [16].

In addition, cultural sensitivity needs to underpin partnerships, where culturally sensitive research may be described as incorporating ‘into its design and implementation the historical context, and cultural experiences, norms, values, beliefs, and behaviors of a distinct ethnic or cultural group.’ [19]^(p. 365)^ Furthermore, it is important to acknowledge and engage with the group’s particular cultural worldview and subsequent discursive practices as expressed in day-to-day living [20].^(p. 136)^

Existing literature on DST’s use with Indigenous communities suggests it might fulfil the imperative for culturally appropriate methods. A study examining the links for Canadian Inuits between climate change and physical, mental, emotional and spiritual health and well-being, found that as a community-driven ‘methodological strategy’ DST can move beyond the limits of narrative research and issues of colonisation associated with western research [21]. The participants themselves stated that the creative process allowed them to ‘explore and share personal ideas, experiences and beliefs’ which ‘was a powerful form of catharsis’.^(p.134)^

Similarly, DST helped Alaskan Native youth ‘re(present)’ their culture and identity within a positive youth development framework [22]. The researchers who initiated the project concluded that creating digital stories helped the youth develop ‘more certain and positive identity formations’, which correlate to more positive health outcomes.^(p.617)^

## Methods

Due to the exploratory nature of the study we adopted a qualitative research design that made use of a constructivist conceptual framework. Constructivism acknowledges the presence of multiple realities; the understanding of phenomena is derived through participants’ subjective views that have been ‘shaped by social interaction with others and from their own personal histories’ [23].^(p.42)^ In tandem, we drew upon principles of Kaupapa Māori research. Kaupapa Māori research is neither a methodology in itself nor an established set of methods. It is an Indigenous approach which at its most fundamental level involves making Māori concerns and priorities the focal point of research that is centred within Māori culture and practice [15]. Specifically, Kaupapa Māori research:

1. ‘gives full recognition to Māori cultural values and systems;
2. is a strategic position that challenges dominant Pakeha [western] constructions of research;
3. determines the assumptions, values, key ideas, and priorities of research;
4. ensures that Māori maintain conceptual, methodological, and interpretive control over research; is a philosophy that guides Māori research and ensures that
5. Māori protocol will be followed during research processes. [24] ^(p.333).^

Points four and five are particularly relevant as this project is a collaborative endeavour between Māori and non-Māori researchers, our research group’s Māori advisory group (rōpū) and the Māori in the community who participated. Regarding point four, central to our perspective was the recognition of our Māori researchers’ (TMM and SB) insider status. In contrast to the non-Māori researchers on the team, whose cultural perspective as outsiders might not ‘reflect the views or reality of the researched’ [24]^(p.335)^ TMM and SB could offer their lived experience of Māori culture. For example, TMM, in consultation with SK, SP, SB and LW, conceptualised how the digital storytelling method might be adapted for use with Māori. TMM suggested the use of the pōwhiri process (explained in Table 1), which is congruent with step five that involves the integration of Māori protocols into the research process. She also initiated a discussion with our Māori advisory group to gauge whether they regarded this project as being in line with Māori priorities for palliative care research.

**Table 1:** The pōwhiri process of engagement

The pōwhiri process of engagement refers to an actual pōwhiri, which is a formal welcome of visitors (manuhiri) by local, Māori hosts (tangata whenua) [36]. The pōwhiri provides a cultural framework to gather people together to carry out cultural customs (such as funerals) or to engage in a discussion or an event of interest and worth to Māori. The pōwhiri begins with a welcome call (karanga) by an esteemed female elder (kuia) and also involves greetings, formal speechmaking by both hosts and visitors as well as a meal afterward [36]. The visitors may be unknown to the hosts. These cultural practices and protocols are useful to help bind the hosts and visitors into a mutually rewarding relationship. In essence, it provides a spiritually safe way for all the people taking part in the meeting to engage with each other and to discuss the topic that underpins the reason for meeting [37].
In addition, the principles underlying the practices and protocols present during the pōwhiri are enacted throughout the research project. For this research, three were integral: *aroha*, *manaakitanga* and *mana*. Aroha may be defined as care and empathy. Manaakitanga is sometimes defined as hospitality, yet also contains a more nuanced meaning. It includes such values as generosity, kindness and a responsibility to care for others [38]. In addition to prestige, mana may refer to the power or authority of an individual. It may also be imagined as a reflection of how the community as a whole regards its wellbeing [39].
Our pōwhiri took place at the University of Auckland’s Māori communal meeting place (marae). The research participants and researchers were the visitors who were welcomed by Māori elders associated with the marae. The pōwhiri process provided a safe space to bind the researchers and Indigenous participants together in preparation for the digital storytelling workshop.

Furthermore, TMM and SB joined in as participants of the workshop, creating their own stories related to end of life issues that their respective extended families (whānau) encountered. Their direct involvement may be considered an expression of *whakawhānaungatanga*, defined as the establishment and continuance of relationships. Originally signifying blood kinship, it now also refers to kinship in a broader sense including the ties of connection and responsibility that such relationship mandates [25]. In the research context, it may be interpreted as walking alongside participants:

‘. . . being involved somatically in the research process; that is, physically, ethically, morally and spiritually and not just as a ‘researcher’ concerned with methodology but as a participant concerned with the well-being of the participants [20].’ ^(p.136)^

### Recruitment

Participants had to have been involved in the care of an older relative (kaumātua) at the end of life to meet the inclusion criteria for the study. TMM began by inviting Māori who had participated in some of her previous research projects and had indicated a willingness to partake in future projects. Such a recruitment process aligns with Kaupapa Māori research principles; it invokes whakawhānaungatanga as a mechanism for engaging with potential participants.

Snowball sampling was then used to enable participants to nominate others they thought would be interested. TMM completed introductory phone calls to determine interest and then met people face to face, emailed or hand-delivered participant information sheets and consent forms. TMM encouraged people to bring members of their extended family for emotional as well as technological support, especially if they were unfamiliar with digital editing software.

Recruitment coincided with New Zealand’s summer holidays, which added to the difficulty in contacting people. Two people who elected to be involved withdrew the week before the workshop due to either a family crisis or illness. Three participants were not recruited until the week of the workshop. This precluded the emailing of critical materials about the workshop, including instructions to come prepared with a story to tell and photographs to illustrate it. Such communication was standard procedure that SK and SP had employed in previous workshops.

### Data collection

We adapted the digital storytelling method described by Lambert [8] to include the pōwhiri before the start of the workshop. The workshop itself was facilitated by SK and SP with the assistance of a technician and research assistant. At the conclusion of the workshop, participants self-completed an anonymous, written questionnaire about their views on the project. The questionnaire consisted mostly of open-ended questions designed to gauge participants’ views on the workshop and their digital story. Open-ended questions were chosen because they allow respondents to answer in their own terms and are less likely to suggest specific kinds of answers [26]. The questionnaire is reproduced in Table 2.

**Table 2:** Questionnaire

Your previous experience using digital media to make videos:
A. extensive experience B. moderate experience C. some experience D. little experience E. no experience
Your relationship to the kaumātua you helped care for _____________________________________________
Questions about the workshop
1. What were your reasons for deciding to participate in the workshop?
2. What did you like most about the digital storytelling workshop?
3. Which part of the digital story telling workshop did you have the most difficulty with?
4. Overall, what did you think of the workshop?
5. What can we do to make this workshop better?
Questions about your digital story
6. Were you pleased with the story you made? Why or why not?
7 If you had someone from your whānau helping you during the workshop, what was it like working with them?
8. Do you think making digital stories is a good way for whānau to tell the community about theirpersonal experiences caring for kaumātua at the end of life? Why or why not?
9. How would you like to see your story used to tell others about caring for kaumātua at the end of life?
10. Please write down here anything else you would like us to know about the workshop or your story.

A questionnaire was chosen as a reporting method rather than a focus group or individual interviews for three reasons. First, the workshop had already imposed an extensive time burden on participants. Second, the researchers (TMM, LW, SK, SB and SP) and participants interacted extensively throughout the 3-day workshop. These interactions allowed the researchers to both observe and discuss with participants their experiences and views on the workshop as it progressed.

Third, because of our interactions, which promoted strong relationships, there was the risk that participants might not give accurate or complete information about issues regarding the workshop or research project they felt critical about. An anonymous questionnaire was employed to mitigate this, and participants did indeed offer information critical of the project.

During the course of the workshop, TMM, LW, SK and SP informally discussed how it was progressing and afterward held a formal debriefing session. The discussions we conducted during the workshop focused on factors impeding or facilitating the completion of the digital stories, especially in relationship to Māori culture, and how individual participants could be better supported to complete their digital stories. In the formal debriefing session we voiced our observations regarding the adaptation of DST for Māori using the pōwhiri process. LW kept extensive field notes on the exchanges.

Regarding the researchers’ observations during the workshop, TMM, LW, SK, SB and SP can be characterised as ‘participant observers’ [27]. Participant observation occurs when researchers partake in the activities of the people being studied [27], with TMM and SB being most engaged as they were themselves creating digital stories and could provide informed feedback due to their dual roles as Māori researchers and participants.

### Participants

Eight participants, seven women and one man attended the 3-day workshop that occurred over one weekend (see Table 3). LW and TM organised the provision of morning and afternoon teas and lunches as well as a light supper at the end of the workshop. Short filmed commentaries with TMM and SB were added after each story several weeks later. Their purpose was instructional, designed to relay cultural implications for health professionals engaging with Māori at the end of life based on issues addressed in the digital stories. TMM and SB discussed, and received approval for, the content of their commentaries with the workshop participants and with the Māori elders who advise the School of Nursing’s Te Ārai Palliative Care and End of Life Research Group. The finished stories are available online for viewing; the link may be found in the reference list under Wharemate et al., 2015) [28]. They are now being used by New Zealand healthcare professionals for training and instructional purposes as well as by School of Nursing staff to teach undergraduate and post-graduate nurses.

**Table 3:** Participant demographics

Age	
40–49	3
50–59	2
60–69	3
Gender	
Female	7
Male	1
Relationship of participant to subject of digital story	
Daughter/Mother	4
Daughter/Father	1
Son-in-law/Mother in law	1
Sister/Brother	1
Sister/Sister	1

### Data analysis

LW and TM undertook an initial thematic analysis of the questionnaires. Thematic analysis is a useful method for researchers who are approaching their topic with a distinct avenue of inquiry in mind (in this case the usefulness of digital storytelling as a method) as it allows them to narrow their focus by scrutinizing particular aspects of the data [29]. TMM, SB, MG (Merryn Gott), SK and SP critiqued the coding scheme; TMM and SB offered suggestions for how to code the data into themes that reflected a Kaupapa Māori research lens. LW and TMM conducted a thematic analysis on LW’s field notes.

## Results

Two main themes were identified during analyses: 1) issues that facilitated digital storytelling’s usefulness as a research method for Māori reporting on end of life caregiving; and 2) issues that hindered this process. The subthemes identified: recruitment, the pōwhiri process, and technology, related to both main themes and are presented in this way below.

### Recruitment

*Facilitated:* TMM’s pre-existing relationships with potential participants made it easier for her to identify those who might be interested in taking part. The whakawhānaungatanga that had already occurred between them helped their willingness to be involved even though it would require a significant time commitment.

*Hindered:* One-on-one recruitment was time-consuming. Participants required extensive engagement. When responding to the question about how the workshop could be improved, they stated:

*‘*Promoting the workshop earlier, having a hui (meeting) to share your expectations and goals for the workshop.’

‘[Need] more info [on] issues regarding [the] gathering of photos, taonga (treasure) stories, music – the process of digital development. Prep work.’

The lack of preparation had a flow-on effect, contributing to time pressures throughout the workshop, especially as participants worked in the digital lab to complete their stories. When answering the question about what they found most difficult about the workshop participants replied, ‘Creating a story in such a short time frame’, ‘Cramming everything into 3 days’ and ‘Gathering all the images in a short time’.

### The pōwhiri process

*Facilitated:* Using the pōwhiri process created space to establish whānaungatanga and this encouraged the emergence of the Māori values of aroha (care and empathy), manaakitanga (hospitality, generosity, kindness, responsibility to care for others) and mana (prestige, power or authority of an individual, wellbeing of the community) which supported and sustained the participants and researchers. All participants responded with enthusiasm about their experience. One regarded it as ‘A+++’. Another replied simply, *‘*Love, love, love this.*’* Regarding the workshop facilitators, participants appreciated their manaakitanga expressed by providing technical and emotional support. For example: ‘To Shuchi, Sarina, Peter (technician), Julie (a research assistant) – great tutors with patience and support. Well done.’ Similarly, another participant reported: ‘I love, love the facilitators, their care, love, and patience was just amazing!’

Being able to bring family members along also fostered a sense of support. Replies to the question addressing what it was like to have a family member helping engendered positive responses:

‘Awesome. It made the process easier. We were able to share, cry and laugh’.

‘Having my sisters to assist in checking to help make decisions along the way was most helpful’.

Crafting digital stories offered participants a new means for upholding the mana of their older relative and craft a treasure for their family:

‘It [making the story] gave me an opportunity to honour our dear mother’.

‘I am very pleased. I’ve created a story for my family’.

*Hindered:* Māori protocol allows for flexible time limits for speakers; they continue until they are finished. This affected our story circle, causing it to run longer than expected thereby contributing to the time pressures already discussed above.

### Using the technology

*Facilitated:* The participants were not experienced using the technology required for the workshop, (Adobe Premiere Elements for MacIntosh v. 13 on the MacIntosh platform). Five participants’ rated themselves as having ‘no experience’ using digital media to make videos, one claimed ‘little experience’ and the two others rated their experience as somewhere between ‘little experience’ and ‘some experience’. No participants considered that they had either ‘moderate’ or ‘extensive’ experience. Even with this lack of experience, some participants rated using the technology as a highlight of the workshop:

‘I loved the experience, [you] can actually learn a lot in 3 days.’

‘Once you know what you’re doing, it gets easier.’

‘Layering the elements together to make the movie/video even though there were frustrations. The challenge and then the sense of achievement.’

*Hindered:* However, as the positive comments indicate, feelings of frustration were intertwined with those of achievement. Using the technology was rated by some as the worst feature of the workshop. It caused fatigue for one participant and made her feel incompetent. Another noted she had ‘worked with Windows for years’, indicating that the unfamiliarity of the MacIntosh platform contributed to the challenge of completing her story. She added that *‘*it takes time to learn [the] Mac version and in such a short time can be frustrating and tiring*.’* Another person stated she had difficulty ‘understanding the technology’. Her remark indirectly referenced the issue of time pressure, which necessitated truncating technical tutorials that SK and SP had included in similar workshops.

## Discussion

This research explores how western research methods might be adapted for use with Indigenous communities. It highlights the need for cultural sensitivity and a participatory research approach. It also exposes the potential for tensions between assumptions inherent in western research methods and the reality of undertaking research with groups who maintain different worldviews. For example, adopting an Indigenous recruitment method allowed us to foreground the relational aspect of the research, (whakawhānaungtanga). By doing so, we were able to keep faith with Māori discursive practices that prioritise relationships [30], a fundamental aspect of identity for Indigenous people [31]. However, we also encountered issues that led to participant dissatisfaction in regards to recruiting.

The negative comments participants offered suggested more than a frustration with a lack of information about the workshop. In line with other Indigenous groups [32, 33], the participant who indicated a preference for a meeting in order to discover our expectations and goals for the workshop was expressing the importance of oral, face-to-face communication, which Māori term ‘kanohi ki te kanohi’. It relates not just to physical presence but to a ‘person’s credibility in words, actions, or intentions’ [34]. ^(p. 441)^ If time had permitted, a preliminary group meeting with participants would have been a way for them to meet the entire research group and thereby support the whakawhānaungatanga that TMM had carried out on the group’s behalf during recruitment.

A meeting could also have served as a culturally sensitive way to dispel any potential mistrust engendered by a research team consisting of non-Indigenous as well as Indigenous researchers. Taking such an opportunity would be important for any project in which the Indigenous ‘researched’ have been adversely affected by western researchers. This point cannot be overstated. Ball and Janyst, quoting an Indigenous Canadian, lay out the issue plainly:

“A lot of us feel we’ve been researched to death, with no benefit to us. Researchers come, they take our stories, take up our time, and leave.

We never see any returns from what we gave.” [35]

As this quote suggests, all too often western research methods ignore the importance of relationships and the necessity to provide the resources required to establish and maintain them.

Indeed, our experience with this DST project points to the need to scrutinise taken-for-granted western research priorities when working with Indigenous people. Different conceptualisations of time, for example, can cause conflicts, which we found when we introduced the pōwhiri process of engagement to our research. Digital storytelling workshops often take place over the course of 3 or 4 days [8] with prescribed time periods set for each task. Our story circle ran longer than expected due to Māori cultural practices, which affected our ability to adhere to our schedule. Yet the benefits to our project of incorporating the pōwhiri process of engagement suggest that it is the schedule that should be adapted rather than the Indigenous protocol. As an example, the participants’ enthusiasm for the workshop and recognition of the researchers’ love and support suggests they were cognisant of a bond that extended beyond those usually created in research situations.

Since the pōwhiri process ritually brings together two peoples (visitors and hosts) to make them one, it creates the possibility for a different type of relationship between researchers and research participants, which may explain the participants’ reaction. Creating space for manaakitanga and aroha, and for an increase in mana, helped fulfil the pōwhiri’s potential to recognise ‘the relative tapu (potentiality for power) and mana (prestige) of all the participants’ [20].^(p.135)^ In other words, the pōwhiri exchanged a hierarchical positioning of people for a collaborative one. Shifting the nature of the relationship is compatible with Māori as well as other Indigenous groups’ views on collaborative, participatory research [24, 32]. It also echoes the tenets of Health Promoting Palliative Care policies, practice and research that prioritises the involvement of communities in research [6].

DST offers marginalised groups a means for expressing alternative voices that can be absent or misrepresented by mainstream discourse. One barrier to their representation is access to and training in the technology necessary to create digital stories [33]. Therefore, providing technical support for Indigenous groups should be emphasised, when required. Fletcher et al. made use of an intergenerational trade-off of skills between Canadian Aboriginal elders and youth to aid story-making [33]. The elders taught the youth about traditional topics such as medicinal plants and cleansing rituals. In turn, the youth showed them how to use the digital equipment required for their stories. As a result, several elders were able to create digital stories, thereby demonstrating that ‘digital stories are a tool that is relevant and accessible across generations.’ ^(p.e185)^ We concur; five of our eight participants were 50 or older and 3 were 60 or older. Though some emphasised their struggle with the technology, others noted positives as well, such as their sense of achievement. With proper support, age does not have to be a barrier to making digital stories. This is important because as populations are ageing, so are family carers. They should not be excluded from telling their stories about providing care at the end of life merely because they might be unaccustomed to the technology required to tell them.

### Limitations

The digital storytelling method as we employed it required people to agree to the public sharing of their stories. This may have limited potential participation by others with stories to tell about caring for an older relative at the end of life who did not wish to make their stories public. In addition, the truncated time period available for recruitment meant participants did not receive information about the workshop ahead of time (either via electronic means or face-to-face) thereby potentially affecting their ability to be adequately prepared for involvement in the research.

## Conclusions

The many disparities amongst countries worldwide means that people face different palliative care and end of life scenarios. As a result, a diversity of responses are called for, which means new research methods are required to explore ways in which care might be most effectively delivered. Digital storytelling is one such method worthy of consideration. In line with Health Promoting Palliative Care initiatives that advocate for participatory, community involvement in research, it provides a means for including Indigenous groups in palliative care research.

## Glossary

Hui: meeting; gathering
Kanohi ki te kanohi: Face to face, in person, in the flesh
Karanga: Formal call; ceremonial welcome call, traditionally the domain of women
Kaumātua: older relatives; older members of the community. A term of respect and esteem
Koha: gift of appreciation
Kōrero: words, talk
Kuia: esteemed older Māori woman; leader
Mana: prestige, power or authority of an individual or a community
Manaakitanga: hospitality including values such as generosity, kindness and a responsibility to care for others
Manuhiri: visitors
Marae: a communal place usually belonging to specific Māori tribes (iwi) with a meeting house and other buildings. (Waipapa Marae where the pōwhiri was held caters for all iwi, as a University based, pan-tribal marae.)
Pākehā: all non-Māori but typically used to designate white New Zealanders
Pōwhiri: a welcoming and hosting encounter between tangata whenua and manuhiri typically on a marae but may occur other places.
Rōpū an advisory group: a community of persons; a committee
Tangata whenua: Māori people of a particular place; or, speaking of Māori in general, the first peoples of New Zealand
Taonga: property, goods, effects, treasure
Tapu: prohibited or sacred, under restriction
Whakawhānaungatanga: establishment and continuance of relationships
Whānau: family including extended family
